# Mechanosensitive ion channel Piezo1 is expressed in antral G cells of murine stomach

**DOI:** 10.1007/s00441-017-2755-0

**Published:** 2017-12-20

**Authors:** Kerstin Lang, Heinz Breer, Claudia Frick

**Affiliations:** 1 Institute of Physiology, University of Hohenheim, Garbenstrasse 30, 70599 Stuttgart, Germany; 2 present address: Department of Microbiome Science, Max Planck Institute for Developmental Biology, Max-Planck-Ring 5, 72076 Tübingen, Germany

**Keywords:** Antrum mucosa, G cells, Piezo1 channel, Enteroendocrine cells, Gastrin

## Abstract

G cells in the antrum region of the murine stomach produce gastrin, the central hormone for controlling gastric activities. Secretion of gastrin is induced mainly by protein breakdown products but also by distensions of the stomach wall. Although G cells respond to protein fragments via distinct chemosensory receptor types, the mechanism underlying G cell activation upon distention is entirely ambiguous. Mechanosensitive ion channels are considered as potential candidates for such a task. Therefore, we explore the possibility of whether Piezo1, a polymodal sensor for diverse mechanical forces, is expressed in antral G cells. The experimental analyses revealed that the vast majority of G cells indeed expressed Piezo1. Within flask-like G cells at the base of the antral invaginations, the Piezo1 protein was primarily located at the basolateral portion, which is thought to be the release site for the exocytic secretion of gastrin. In the spindle-like G cells, which are oriented parallel to the invaginations, Piezo1 protein was restricted to the cell body where the hormone was also located, whereas the long processes appeared to be devoid of Piezo1 protein. Our results suggest that mechanosensitive channels such as Piezo1, located in close proximity to hormone-release sites, enable G cells to respond directly to antrum distensions with gastrin secretion.

## Introduction

Endocrine cells in the gastrointestinal tract respond to a wide variety of stimuli including nutrients and non-nutrient factors such as pH and mechanical stimuli. In the stomach, a particularly important population of enteroendocrine cells are the G cells, which produce gastrin, the central hormone for controlling gastric activities (Dockray et al. 2001). G cells are almost exclusively located in the antral gastric mucosa and release gastrin in response not only to the luminal presence of partially digested proteins but also to the distension of the stomach wall (Schiller et al. 1980; Schubert and Makhlouf 1993). The idea that luminal nutrients might act directly on the cells (Lichtenberger 1982; DelValle and Yamada 1990) is strongly supported by recent studies demonstrating the expression of chemosensory receptors for protein breakdown products and amino acids including GPR92, GPRC6A and CaSR (Haid et al. 2012). The mechanisms leading to the stimulation of G cells by antrum distension remain elusive. Previous studies have indicated that distension-induced responses of G cells are mediated by neuronal reflexes and, thus, require an intact antral innervation (Schubert and Makhlouf 1993). However, a more recent investigation revealed that, following antral denervation, the sensitivity of gastrin cells to distension is even increased (Higham et al. 1997). These findings imply that a mechanism exists for the distension-evoked release of gastrin and that this mechanism is independent of the antral innervation. Thus, speculation that G cells possess intrinsic mechanosensitivity is tempting (Higham et al. 1997). This notion suggests that G cells closely resemble enterochromaffin (EC) cells, which are well known as being stimulated to release 5-hydroxytryptamine by mechanical forces and which are considered important mechanosensors of the gastrointestinal epithelium (Bertrand 2004). So far, any evidence for the presence of mechanosensitive proteins that could render G cells mechanoresponsive is lacking. The recently discovered Piezo channels are increasingly recognized as significant contributors to cellular mechanosensitivity. Piezo1 and Piezo2 are nonselective cationic ion channels that are directly activated by mechanical forces and have well-defined biophysical properties (Coste et al. 2010; Bagriantsev et al. 2014). Whereas Piezo2 is predominantly found in sensory tissues, such as dorsal root ganglia sensory neurons and Merkel cells and is narrowly tuned to detect mechanical touch specifically, Piezo1 is considered as a polymodal sensor of diverse mechanical forces and is primarily expressed in nonsensory tissues exposed to fluid pressure and flow, e.g., kidneys and blood cells (Wu et al. 2017). Therefore, in this study, we set out to answer the question as to whether the mechanosensitive ion channel Piezo1 is expressed in enteroendocrine cells of the gastric mucosa.

## Materials and methods

### Mice

Analyses were performed with the wild-type mouse strain C57/BL6J purchased from Charles River (Sulzfeld, Germany) and the BAC-transgenic mouse line mGas-EGFP, which expresses enhanced green fluorescent protein (EGFP) under the control of the gastrin promoter (Takaishi et al. 2011). Mice were housed with a 12-h light/dark cycle in groups or individually at the Central Unit for Animal Research at the University of Hohenheim and had access to food and water ad libitum. Experiments were carried out in accordance with the Council Directive 2010/63EU of the European Parliament and the Council of 22 September 2010 on the protection of animals used for scientific purposes. The work was approved by the Committee on the Ethics of Animal Experiments at the Regierungspräsidium Stuttgart (V318/14 Phy) and the University of Hohenheim Animal Welfare Officer (T125/14 Phy, T126/14 Phy).

### Reverse transcription plus polymerase chain reaction

Reverse transcription plus polymerase chain reaction (RT-PCR) amplification was conducted by using normalized cDNA from the antrum, antrum control lacking reverse transcriptase and the jejunum. PCR amplifications were performed with the following primer combination: Piezo1 forward, 5′- ACT TTG CCC TGT CCG CCT A-3′; Piezo1 reverse, 5′-GAA GAA GCC CCG CAC AAA C-3′. RT-PCR was carried out by using Phusion PCR Enzyme Mix (New England BioLabs, Ipswich, Mass., USA) and a Peqstar thermo cycler (Peqlab, Erlangen, Germany). For amplification, the following PCR cycling profile was used with annealing temperatures adjusted to the employed primer combination and optimized numbers of amplification cycles, as specified in the following: one cycle: 2 min at 98 °C; 20 cycles: 30 s at 98 °C, 40 s at 67 °C with −0.5 °C per cycle, 1 min 10 s at 72 °C; 25 cycles: 30 s at 98 °C, 40 s at 57 °C, 1 min 10 s at 72 °C; and one cycle: 5 min at 72 °C.

PCR products were run on 1% agarose gels containing ethidium bromide. Amplification of a 204-bp fragment from the mouse housekeeping control gene ribosomal protein l8 (RPL8) was used as a control to confirm equal quantities of the cDNA preparations.

### In situ hybridization

Animals were killed by CO_2_ asphyxiation and subsequent decapitation. The abdomen was opened via an incision through the integument and the abdominal wall. After the stomach was taken out, the fundic tissue was cut off. Afterwards, the stomach was opened along the lesser curvature and washed with 1 × phosphate-buffered saline (PBS). The antral tissue and the adjacent corpus, pyloric and duodenal tissue were embedded in Tissue Freezing Medium (Leica Microsystems, Bensheim, Germany) and quickly frozen on liquid nitrogen. Cryosections (8 μm) were generated by using a CM3050S cryostat (Leica Microsystems) and were attached to Polysine slides (Menzel Gläser, Braunschweig, Germany).

Digoxigenin-labeled antisense riboprobes were generated from partial cDNA clones in pGem-T plasmids encoding Piezo1 (NCBI accession number NM_001037298.1) by using the T7/SP6 RNA transcription system (Roche Diagnostics, Mannheim, Germany) as recommended by the manufacturer. The specificity of the signals was confirmed by control experiments with corresponding sense probes. After fixation in 4% paraformaldehyde/0.1 M NaHCO_3_, pH 9.5, for 45 min at 4 °C, sections were washed in 1 × PBS for 1 min at room temperature, incubated in 0.2 M HCl for 10 min and in 1% Triton X-100/1 × PBS for 2 min and washed again twice in 1 × PBS for 30 s. Finally, sections were incubated in 50% formamide/5 × SSC (0.75 M NaCl, 0.075 M sodium citrate, pH 7.0) for 5 min. Then tissue was hybridized in hybridization buffer (50% formamide, 25% H_2_O, 25% Microarray Hybridization Solution Version 2.0 [GE Healthcare, Freiburg, Germany]) containing the probe and incubated in a humid box (50% formamide) at 65 °C overnight.

After slides were washed twice in 0.1 × SSC for 30 min at 65 °C, they were treated with 1% blocking reagent (Roche Diagnostics, Mannheim, Germany) in TBS (100 mM TRIS, 150 mM NaCl, pH 7.5) with 0.3% Triton X-100 for 30 min at room temperature (in a humid box) and incubated with an anti-digoxigenin alkaline-phosphatase-conjugated antibody (Roche Diagnostics) diluted 1:750 in TBS with 0.3% Triton X-100/1% blocking reagent at 37 °C for 30 min. After two washes in TBS for 10 min, hybridization signals were visualized by using 0.0225% NBT (nitroblue tetrazolium) and 0.0175% BCIP (5-brom-4-chlor-3-indolyl phosphate) dissolved in DAP buffer (100 mM TRIS, pH 9.5, 100 mM NaCl, 50 mM MgCl_2_) as substrates. Sections were mounted in MOWIOL (10% polyvinyl alcohol 4–88 [Sigma], 20% glycerol in 1 × PBS).

### Tissue preparation for immunohistochemistry

Animals were killed by CO_2_ asphyxiation and tissues were fixed by using 4% paraformaldehyde and 0.1% glutaraldehyde perfused via the vascular system. An incision through the integument and abdominal wall was made and the rib cage was carefully opened to expose the heart. To prepare the mouse for the perfusion, a needle was introduced into the left ventricle and an incision to the right atrium was made. Via the perfusion needle, first 10 ml ice-cold 1 × PBS (0.85% NaCl, 1.4 mM KH_2_PO_4_, 8 mM Na_2_HPO_4_, pH 7.4) was applied followed by 3 × 10 ml ice-cold 4% paraformaldehyde (in 150 mM phosphate buffer, pH 7.4) + 0.1% glutaraldehyde. After perfusion, the abdomen was opened and the stomach was removed. Next, the fundic tissue was cut off and the stomach was opened along the lesser curvature and washed with ice-cold 1 × PBS. The antral tissue and the adjacent corpus and the pyloric and duodenal tissue were mounted on a piece of rubber and immersion-fixed for 24 h in 4% paraformaldehyde/1 × PBS at a ratio of 1:1. After fixation, the tissue was cryoprotected by incubation in 25% sucrose overnight at 4 °C. Finally, the tissue was embedded in Tissue Freezing Medium (Leica Microsystems, Bensheim, Germany) and quickly frozen on dry ice or liquid nitrogen. Cryosections (8 μm) were generated by using a CM3050S cryostat (Leica Microsystems) and attached to Superfrost Plus microscope slides (Menzel Gläser, Braunschweig, Germany).

### Immunohistochemistry

Cryosections were air-dried, rinsed in 1 × PBS for 10 min at room temperature and blocked in 0.5% Triton X-100 in 1 × PBS containing 10% normal donkey serum (NDS; Dianova, Hamburg, Germany) for 30 min at room temperature. For single- and double-labeling experiments, primary antibodies were diluted in 0.5% Triton X-100 in 1 × PBS containing 10% NDS. Antibodies were used in the following dilutions: rabbit anti-Piezo1 (ab128245, abcam, Cambridge, United Kingdom) at 1:50 and guinea pig anti-gastrin (BP5046, Acris Antibodies, Herford, Germany) at 1:1000. Blocked sections were incubated with the diluted primary antibodies overnight at 4 °C. For the blocking experiments, specific Piezo1 immunizing peptide (ab133015, abcam) was added to the Piezo1 antibody. After washes in 1 × PBS, the bound primary antibodies were visualized by using donkey anti-rabbit IgG (H + L) secondary antibody bound to Alexa Fluor 568 (Invitrogen™, Fisher Scientific, Göteborg, Sweden) and goat anti-guinea pig IgG (H + L) secondary antibody bound to Alexa Fluor 488 (Jackson Immunoresearch, Newmarket, Suffolk, UK), diluted in 1 × PBS with 0.5% Triton X-100 containing 10% NDS for 2 h at room temperature. After three rinses for 5 min in 1 × PBS, tissue sections were counterstained with 4′,6-diamidino-2-phenylindole (DAPI; 1.0 μg/ml, Sigma Aldrich, Schnelldorf, Germany) or propidium iodide (1.0 mg/ml, Thermo Fisher, Darmstadt, Germany) ata 1:1000. Following incubation for 3 min at room temperature, sections were rinsed with double-distilled water and mounted in MOWIOL (10% polyvinyl alcohol 4–88 (Sigma), 20% glycerol in 1 × PBS). No immunoreactivity could be observed when the primary antibodies were omitted.

### Microscopy and photography

Immunhistochemical staining was documented by using a Zeiss Axiophot microscope (Carl Zeiss MicroImaging, Jena, Germany). Images were adjusted for contrast in AxioVision LE Rel. 4.3 (Carl Zeiss MicroImaging).

### Cell counting

For determination of the number of Piezo1-immunoreactive G cells in antral invaginations, EGFP-positive cells were counted manually on longitudinal sections through the antral mucosa. Tissue sections were counterstained with DAPI to visualize all cells. The number of all EGFP-positive G cells of randomly selected sections was counted, together with the number of Piezo1-positive cells. For the quantification of Piezo1-expressing G cells, three mice were used and a total of 87 (8-μmthick) sections were analyzed.

## Results

### Expression of Piezo1 channels in antrum of murine stomach

**Fig. 1:**
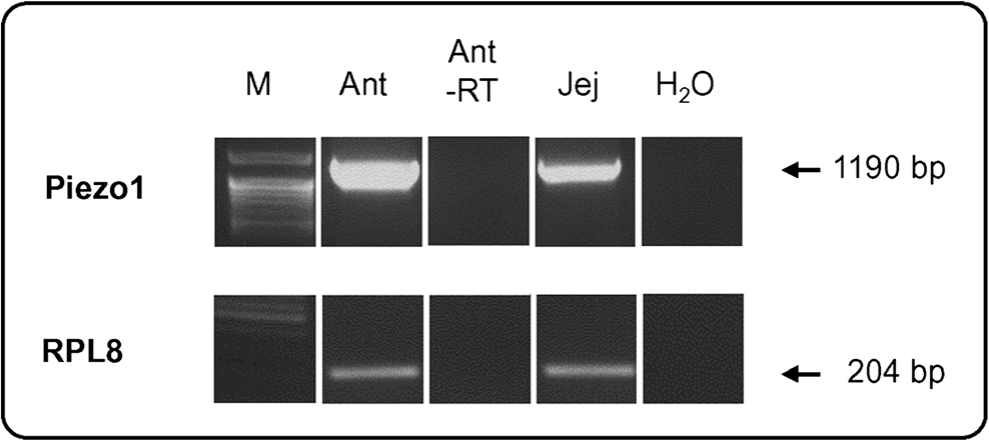
Identification of mRNA for Piezo1 and the ribosomal protein L8 (*RPL8*) in the gastrointestinal tract. Semi-quantitative reverse transcription plus polymerase chain reaction experiments performed with primer pairs specific for Piezo1 (1190 bp) and RPL8 (204 bp). Amplicons for Piezo1 and RPL8 were obtained by using normalized cDNA from the antrum (*Ant*) and jejunum (*Jej*). Whereas cDNA from the antrum and jejunum yielded comparable bands for RPL8, cDNA from the antrum yielded a stronger band for Piezo1 compared with cDNA from the jejunum. No bands were observed in antrum controls lacking reverse transcriptase (*Ant -RT*) and water controls lacking template (*H*_*2*_ *O*)

**Fig. 2:**
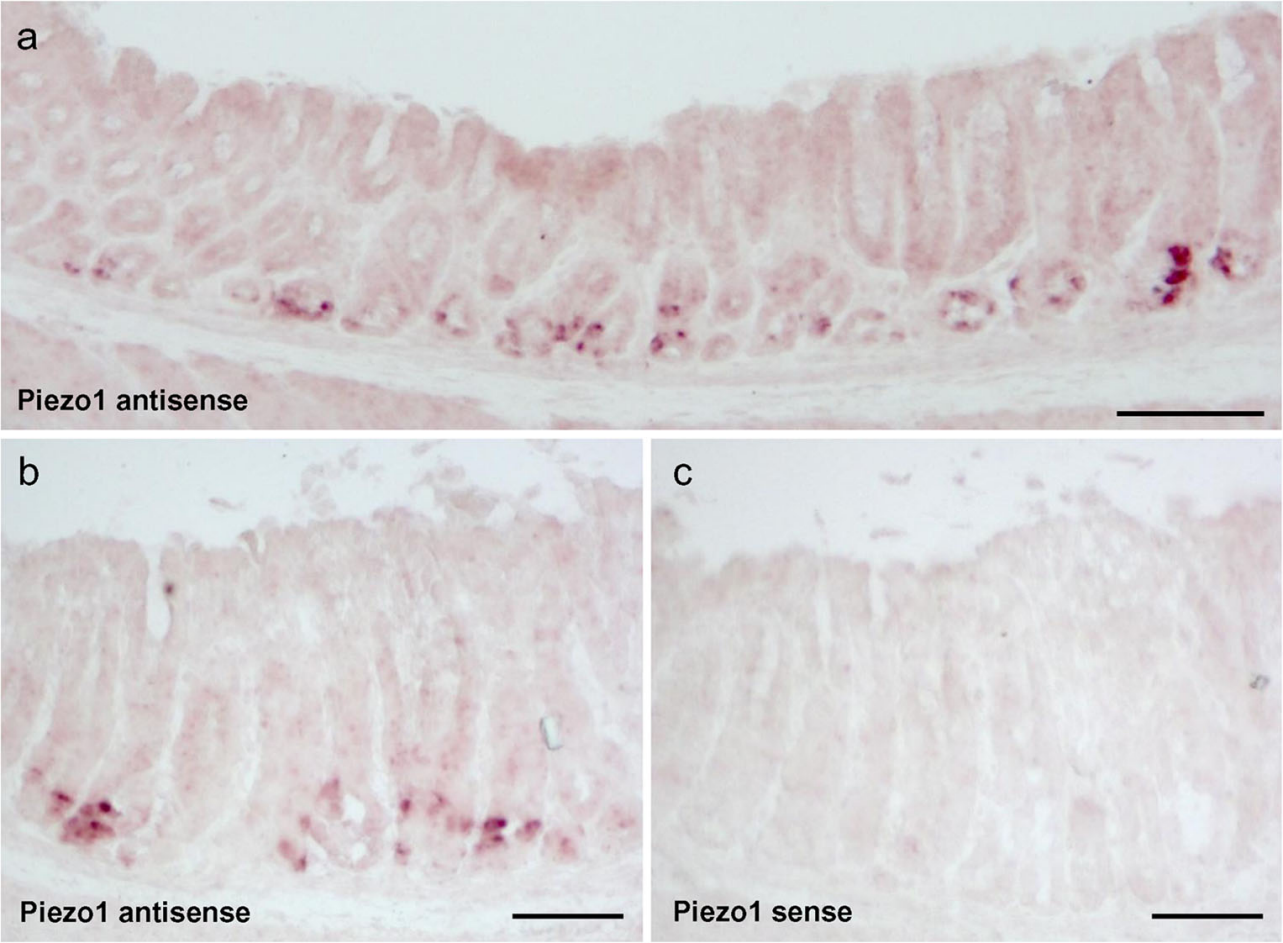
Localization of mRNA for Piezo1 in the antral mucosa. In situ hybridization experiments performed with probes specific for Piezo1. **a** Signals in the antral mucosa were obtained by using Piezo1 antisense probes. **b** Whereas Piezo1 antisense probes generated strong signals in the basal region of the antrum, no signals were obtained when Piezo1 sense probes were used (**c**). *Bars* 200 μm (**a**), 100 μm (**b**, **c**)

**Fig. 3:**
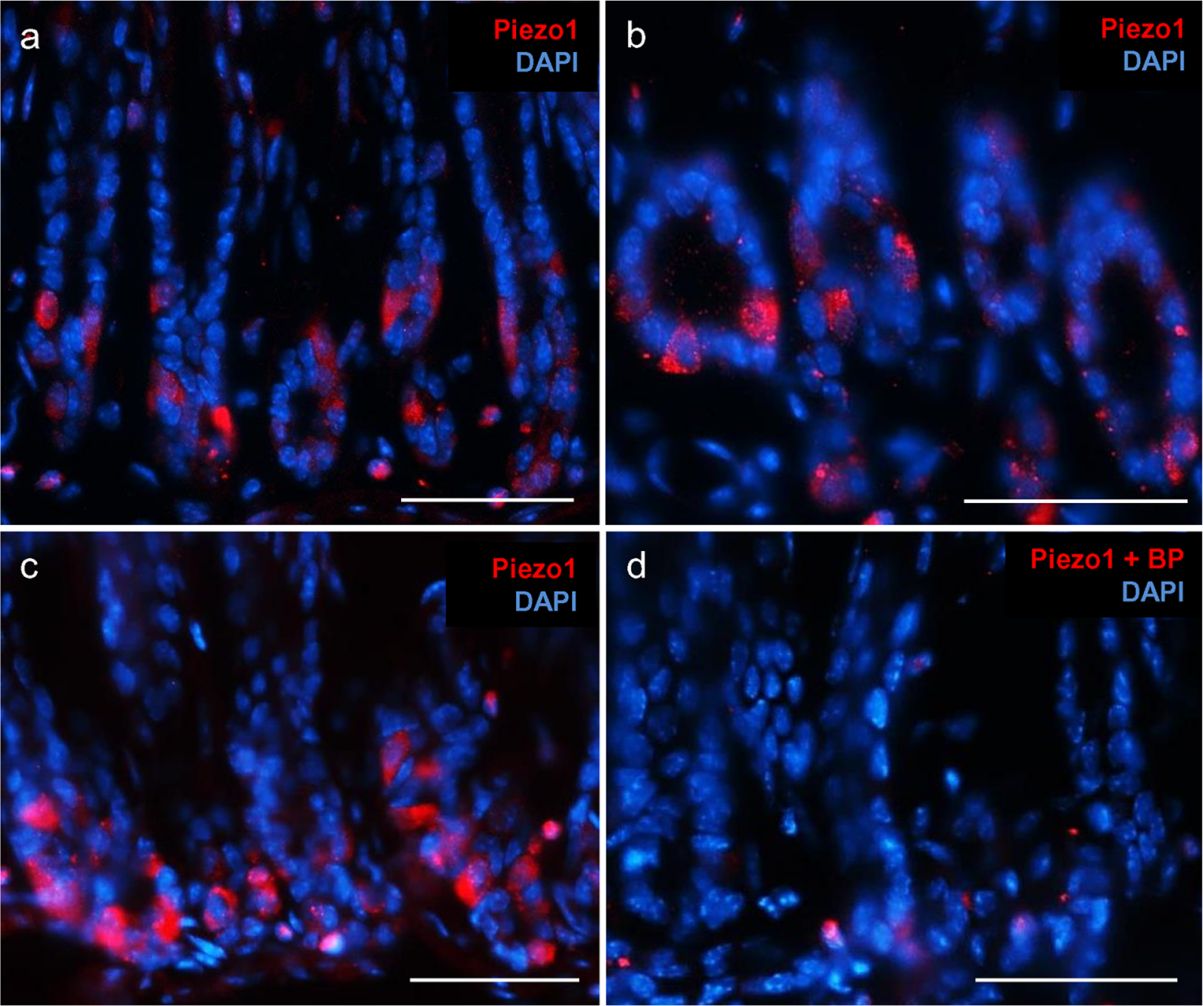
Localization of Piezo1 protein in the antral mucosa of mice. Immunohistochemical staining was performed with an antibody against Piezo1 (*red*). Nuclei were counterstained with 4′,6-diamidino-2-phenylindole (*DAPI*, *blue*). **a** Antral invaginations were visible in longitudinal sections. Piezo1-positive cells were mostly located in the basal region. **b** The round structure of the antral invagination was visible in horizontal sections. Piezo1-positive cells formed part of the epithelium. **c** In control experiments with the antibody without the blocking peptides, distinct cells in the antral mucosa were labeled. **d** On sections incubated with the antibody in combination with the specific blocking peptide (*BP*), the labeling was almost completely abolished. *Bars* 50 μm

Since G cells are almost exclusively located in the antrum region of the stomach (Frick et al. 2017), we initiated the study by analyzing the antrum of a murine stomach for expression of the mechanosensitive channel Piezo1 by semi-quantitative RT-PCR experiments. Samples of cDNA from the antrum and, for comparison, from the small intestine of adult mice were analyzed by using primer pairs specific for Piezo1 and for the housekeeping gene RPL8. Whereas similar amplicons were obtained with RPL8 primers, the use of primer pairs for Piezo1 resulted in a strong band with cDNA from the antrum and a smaller band with cDNA from the jejunum (Fig. 1), indicating that a significant amount of mRNA for Piezo1 was present in the antrum. In order to explore the location in the antrum in which mRNA for Piezo1 was expressed, specific probes were generated for in situ hybridization experiments. Incubation of tissue sections of the antrum mucosa with Piezo1 antisense probes resulted in the labeling of cells mostly located in the basal region of the antrum (Fig. 2a). In contrast to the strong signals obtained with antisense probes (Fig. 2b), no labeling was obtained with sense probes (Fig. 2c). These results indicated that most of the cells that expressed the Piezo1 gene were located in the basal region of the antral mucosa. In order to explore whether the Piezo1 ion channel protein was indeed present in antrum mucosa cells, specific antibodies for Piezo1 were employed in immunohistochemical approaches. Longitudinal and horizontal sections of the antral mucosa were analyzed and counterstained with DAPI to visualize the cellular organization of the antral invaginations. The results depicted in Fig. 3. indicate that distinct cells were specifically immunolabeled by the Piezo1 antibody. In longitudinal sections, the labeled cells were visible primarily at the base of the antral invagination (Fig. 3a). In horizontal sections, the invaginations of the antral glands were visible and the labeled cells formed an integral part of the lining epithelium (Fig. 3b). In order to confirm the specificity of the antibody labeling, control experiments were performed with antibodies in combination with the corresponding blocking peptide. The results are documented in Fig. 3c, d; the labeling of epithelial cells with the Piezo1 antibody, as seen in Fig. 3c, was abolished in sections that were incubated with the Piezo1 blocking peptide together with the antibody. These results confirmed the specificity of the Piezo1 antibody labeling (Fig. 3d).

### Expression of Piezo1 in G cells

**Fig. 4:**
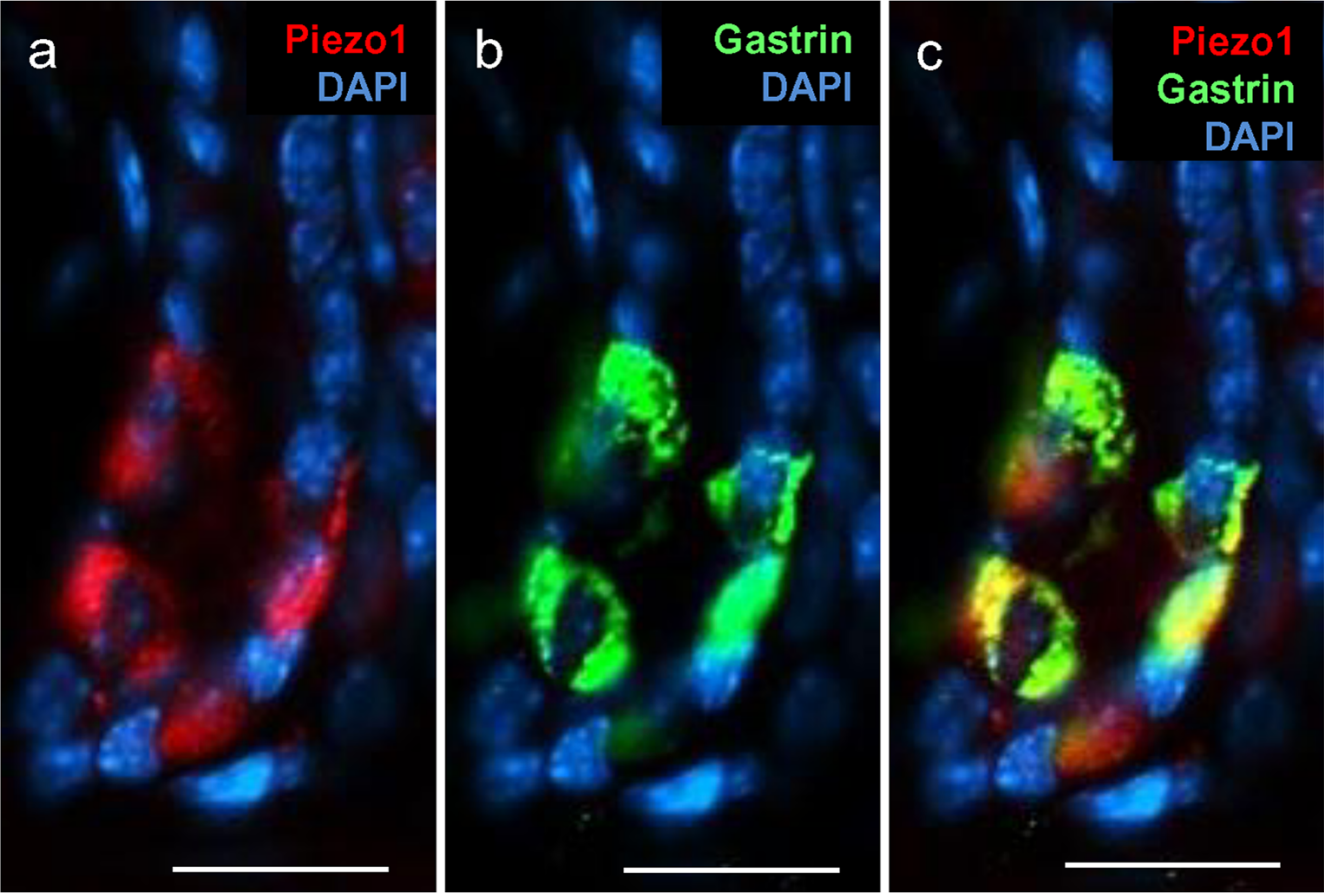
Localization of Piezo1 protein in gastrin-positive cells of the antral mucosa. Double-immunohistochemical staining of longitudinal sections from the antrum mucosa with antibodies for Piezo1 (*red*) and gastrin (*green*). Nuclei were counterstained with DAPI (*blue*). **a** Specific Piezo1 antibody labeled distinct epithelial cells at the base of the invaginations. **b** Specific antibody for gastrin labeled antral epithelial cells. **c** Overlay of **a**, **b**. *Bars* 25 μm

Given that the base of antral invaginations harbors several types of enteroendocrine cells, we hypothesized that some of these enteroendocrine cells expressed Piezo1. Since gastrin-producing G cells are the major enteroendocrine cell type in the antrum, we focused our investigations primarily on G cells. Double-labeling approaches were performed by using a specific antibody against gastrin to visualize G cells and a specific antibody against Piezo1. The results are depicted in Fig. 4. At the base of the antral invaginations, distinct cells in the epithelium were labeled by Piezo1 (Fig. 4a) and gastrin antibodies (Fig. 4b). The overlay revealed that cells immunoreactive for gastrin were also labeled by the Piezo1 antibody (Fig. 4c). These results indicated that Piezo1 was expressed in gastrin-secreting G cells.

**Fig. 5:**
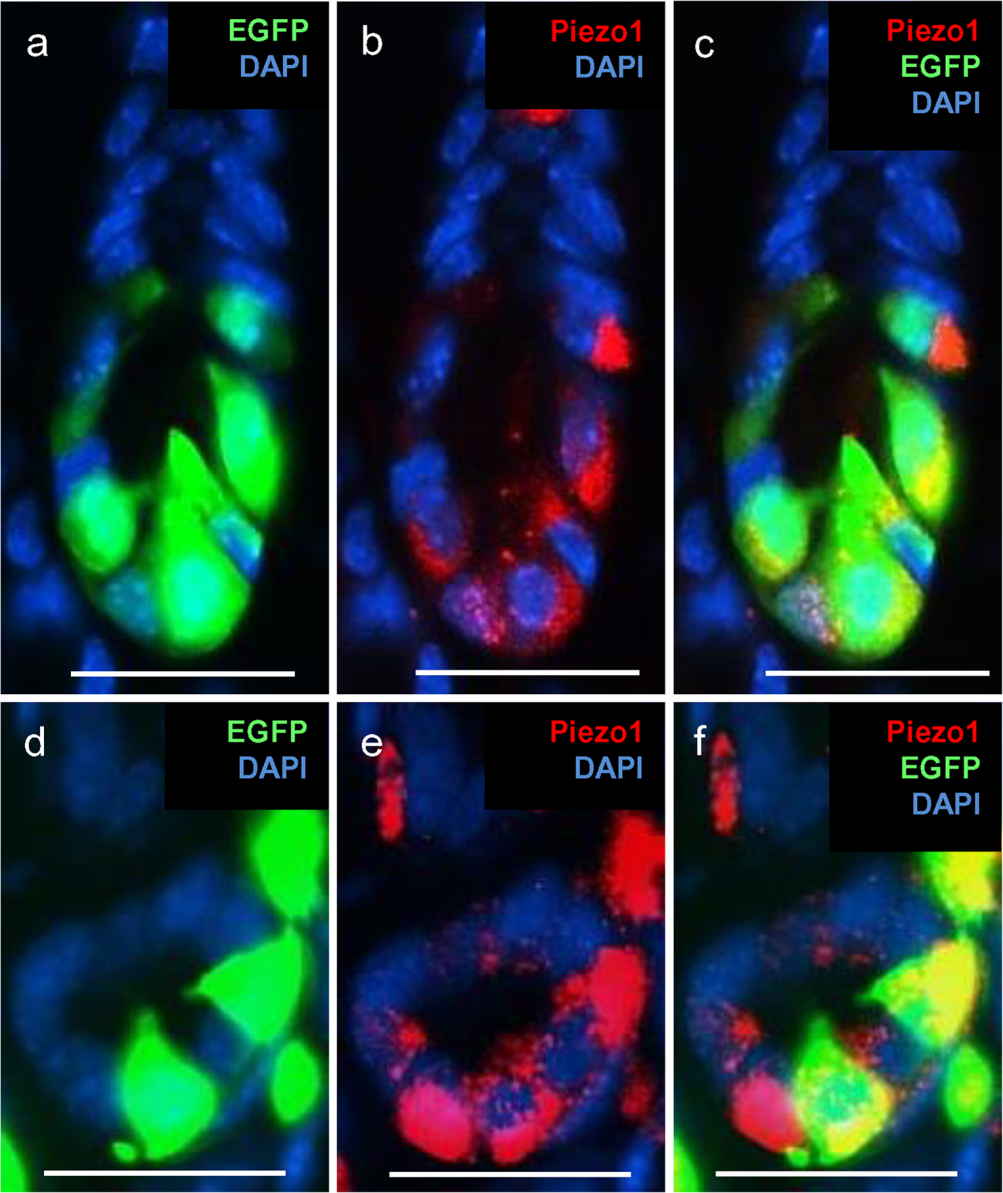
Localization of Piezo1 protein in G cells of the transgenic mGas-EGFP mouse line in longitudinal (**a-c**) and horizontal (**d-f**) sections. Immunohistochemical staining was performed with a specific antibody for Piezo1 (**red**). Nuclei were counterstained with DAPI (*blue*). **a**, **d** Intrinsic enhanced green fluorescent protein (EGFP) fluorescence allowed the identification of G cells and the visualization of their morphology. **b**, **e** Piezo1 immunoreactivity of EGFP-positive G cells was restricted to the basal part of the cell body. **c**, **f** EGFP-positive G cells were also immunopositive for Piezo1. *Bars* 25 μm

**Fig. 6:**
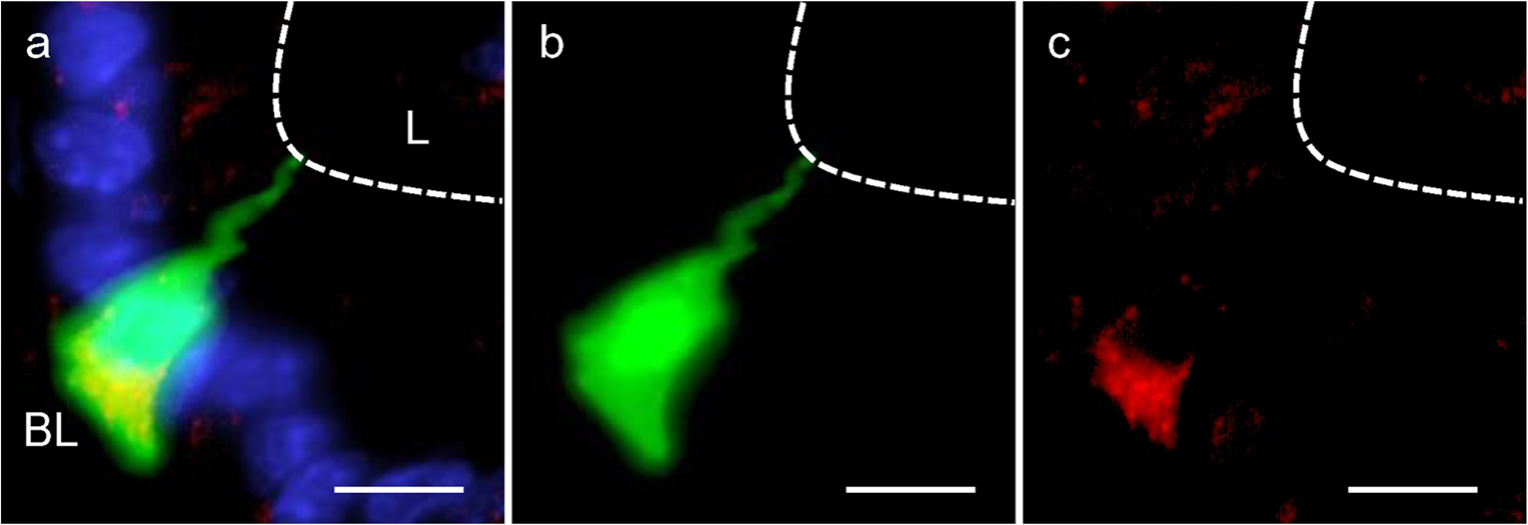
Localization of Piezo1 protein in G cells is restricted to the basal part of the cell body. Immunohistochemical staining was performed with a specific antibody against Piezo1 (*red*). Nuclei were counterstained with DAPI (*blue*). **a** The EGFP-positive G cell was immunopositive for Piezo1. **b** Intrinsic EGFP fluorescence allowed the identification of G cells and the visualization of their morphology. **c** Piezo1 immunoreactivity of EGFP-positive G cells was restricted to the basal part of the cell body (*dashed line* membrane border to lumen, *BL* basolateral side, *L* lumen). *Bars* 25 μm

With a view of unraveling the possible function of Piezo1 ion channels in G cells, we considered it to be of great interest to explore the exact location of the mechanosensory protein in these hormone-secreting cells. Conventionally, G cells have been visualized by labeling this cell type immunohistochemically by using antibodies against gastrin. Visualization of gastrin, however, does not reveal the entire cell morphology as only compartments with sufficient levels of gastrin are visible, such as the basolateral portion of the cells. To overcome this obstacle, we used a transgenic mGas-EGFP mouse line (Takaishi et al. 2011) in which EGFP is expressed under the promoter of the gastrin gene. The marker EGFP is distributed throughout the cells and thus allows the visualization of the complete morphology of the G cells in the antrum of the mouse stomach (Frick et al. 2016). On longitudinal sections through the antrum mucosa of the transgenic mouse line, several green fluorescent cells were visible at the base of the invaginations because of the expressed EGFP (Fig. 5a). Their flask-like shape with a large base and a prominent apical pole projecting towards the lumen was also visible in cross-sections (Fig. 5d). With a specific antibody against Piezo1, distinct cells were labeled in these sections (Fig. 5b, e). The overlays made it immediately apparent that the green cells were also immunopositive for Piezo1 (Fig. 5c, f); however, not all Piezo1 cells seemed to be EGFP-positive. Upon closer inspection, we noticed that Piezo1 immunoreactivity was not equally distributed throughout the cell but rather was concentrated at the base of the green-labeled cells. This aspect was analyzed in more detail. A typical result is presented in Fig. 6. In this tissue section immunolabeled for Piezo1, an EGFP-labeled cell is visible integrated into the epithelial lining of the lumen (visible by DAPI staining) with a long apical process. Yellow labeling attributable to the overlay of EGFP (green) and Piezo1 immunoreactivity (red) is seen only at the base of the cell (Fig. 6a). The restricted localization of the Piezo1 immunoreactivity at the basolateral portion of the cell became even more obvious on single channel images (Fig. 6c). Thus, Piezo1 seems to be located primarily at the site of G cells where typically hormone granules are concentrated and the hormone is released.

**Fig. 7:**
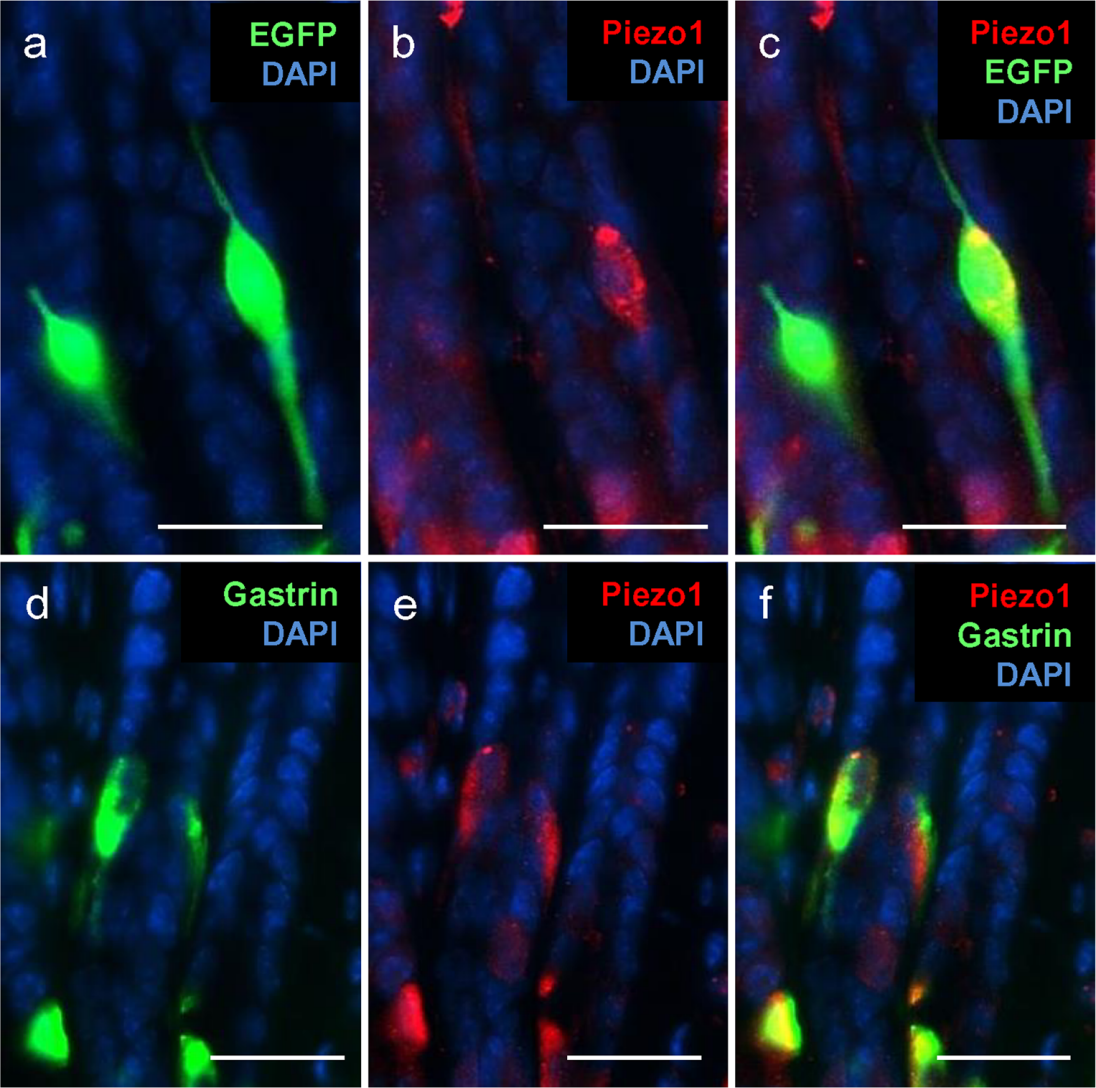
Localization of Piezo1 protein in spindle-like G cells of the transgenic mGas-EGFP (**a-c**) and wild-type (**d-f**) mouse line. Immunohistochemical staining was performed with specific antibodies against Piezo1 (*red*) and gastrin (*green*). Nuclei were counterstained with DAPI (*blue*). **a** Intrinsic EGFP fluorescence allowed the identification of spindle-like G cells and the visualization of their morphology. **b** Piezo1 immunoreactivity of EGFP-positive G cells was restricted to the cell body. **c** Spindle-like G cells were also immunopositive for Piezo1. **d** Gastrin immunoreactivity was found in spindle-like G cells of the upper region of the antral crypts. **e** Labeling of upper epithelial cells in the antrum was obtained by using a specific Piezo1 antibody. **f** The merged image indicates that gastrin-immunoreactive cells in the upper region were also immunopositive for Piezo1. *Bars* 25 μm

In the course of a previous study, Frick et al. (2016) found that, in the upper region of antral invaginations, spindle-like G cells are located with long processes that extend vertically along the invaginations. In longitudinal sections of the antral mucosa from mGas-EGFP mice, EGFP-labeled spindle-like cells were visible with long processes parallel to the invagination (Fig. 7a). An analysis of these sections with a Piezo1 antibody resulted primarily in the staining of the cell bodies (Fig. 7b). This segregated labeling was more clearly seen in the merged images, in which the double-labeling (yellow) was only seen in the cell body, whereas the long processes, made visible by EGFP, seemed to be devoid of any Piezo1 immunoreactivity (Fig. 7c). Labeling of these spindle-like G cells with the gastrin antibody resulted in the staining of the cell body, indicating that the hormone seemed to be mainly restricted to the cell soma. The incubation of the same section with Piezo1 antibodies led to the labeling of the cell bodies (Fig. 7e). In the merged image depicted in Fig. 7f, the yellow labeling indicating the localization of gastrin and Piezo1 was restricted to the cell soma, thus fortifying the results mentioned above. These observations indicated a segregated distribution of Piezo1 in both G cell types and suggested a localization of the channel protein close to the hormone-release sites.

### Quantification of Piezo1-expressing G cells

**Fig. 8:**
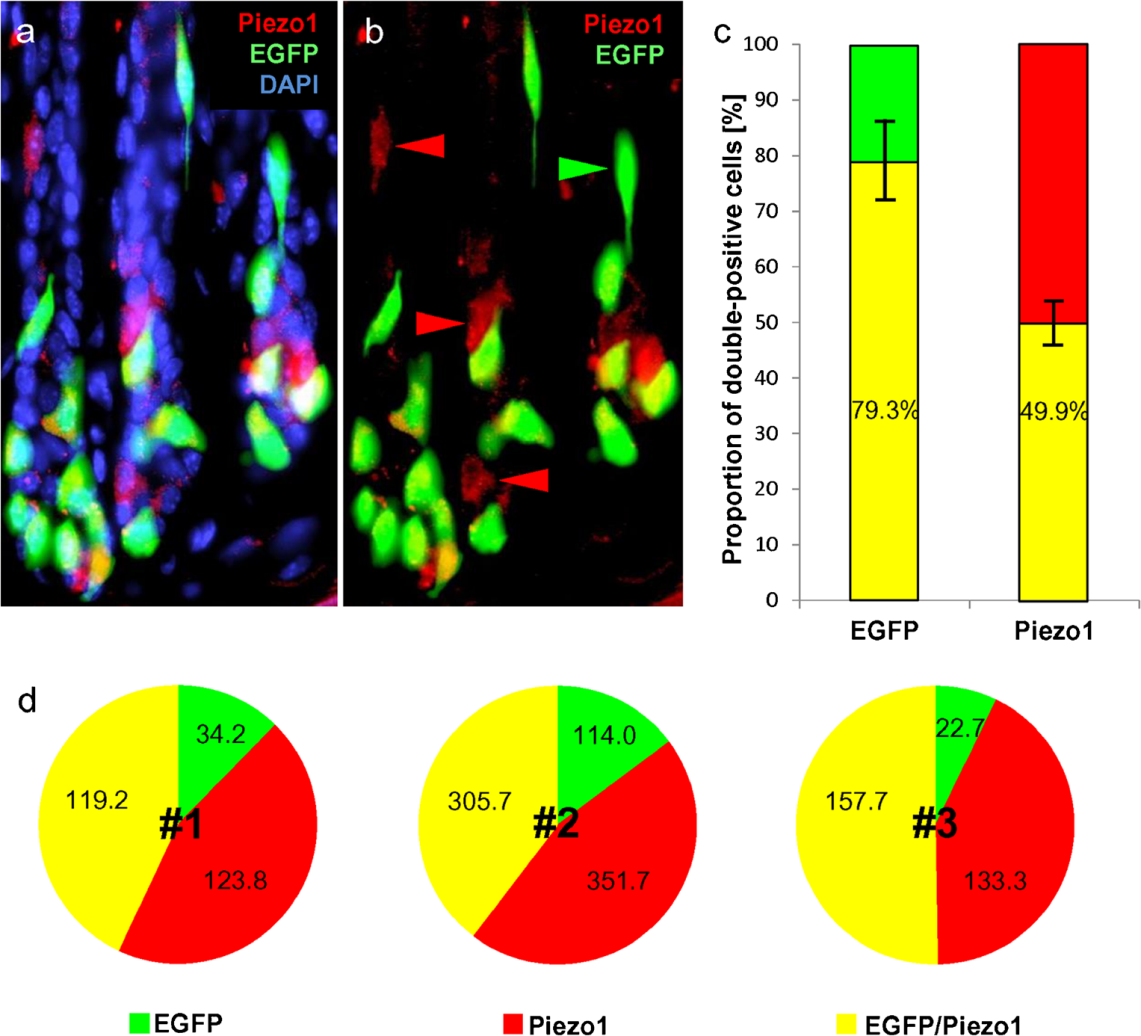
Quantification of EGFP- and Piezo1-positive cells in the transgenic mGAS-EGFP mouse line. Immunohistochemical staining was performed with an antibody for Piezo1 (*red*). Intrinsic EGFP fluorescence marked antral G cells (*green*). Nuclei were counterstained with DAPI (*blue*). **a** Antibody labeling of epithelial cells in the antrum was obtained by using a specific Piezo1. **b** The vast majority of EGFP-positive G cells were found to be immunopositive for Piezo1. Moreover, some G cells were not immunopositive for Piezo1 (*green arrowhead*) and some Piezo1-immunopositive cells lacked EGFP fluorescence (*red arrowheads*). **c** Quantification of EGFP- and/or Piezo1-positive cells revealed that 79.3% (±7.4%) EGFP-positive G cells were Piezo1-immunopositive and that 49.9% (±3.9%) Piezo1-immunopositive cells were EGFP-positive. **d** Absolute numbers of EGFP-positive and/or Piezo1-immunopositive cells were counted on tissue sections of three mice (*n* = 3)

To determine the proportion of G cells that indeed express Piezo1, the number of EGFP-labeled cells, of Piezo1-reactive cells and of cells positive for both EGFP and Piezo1 was determined on tissue sections as depicted exemplarily in Fig. 8a, b. We found that about 80% (79.3% ±7.4%) of the EGFP-positive G cells were Piezo1-positive. On the other hand, almost half (49.9% ±3.9%) of the Piezo1-positive cells were also EGFP-positive (Fig. 8c). For comparison, the absolute number of these three cell types determined for three individual mice are documented in Fig. 8d. The data indicate that the total number of labeled cells varies considerably between the three mice; however, the proportion of the various cell populations is almost identical in each mouse.

## Discussion

The enteroendocrine G cells in the antral region of the stomach release gastrin, the central hormone for controlling the activities of the stomach (Dockray et al. 2001). Accordingly, the secretory activities of G cells are fine-tuned by a variety of parameters, including antrum distension. A direct response of G cells to mechanical stimuli is thought to be mediated by mechanosensitive ion channels, which are transmembrane proteins forming ion conduction pores gated by mechanical forces (Árnadóttir and Chalfie 2010). As part of a search for candidate mechanosensitive ion channels in G cells, this study demonstrates that the mechanosensory channel Piezo1 is expressed in the antrum region of the murine stomach, most notably in gastrin-releasing G cells within the antral invaginations. The expression of Piezo1 appears to be quite specific for enteroendocrine cells, since no indication has been obtained of Piezo mRNA and protein in the adjacent epithelial cells (enterocytes). By means of a transgenic mGas-EGFP mouse line (Takaishi et al. 2011), which allowed us to visualize the entire morphology of G cells, we determined that the Piezo1 protein was not equally distributed throughout the cell but rather was concentrated at the basolateral region of the cells. This observation is of particular interest, since gastrin is stored in secretory granules present along the basolateral portion of the cell and is released into the circulation at the basolateral membrane in response to appropriate stimuli (Smith and Morton 2010). Furthermore, the exocytic release process is strictly calcium-dependent (Harty and Maico 1981). Thus, the pore-forming Piezo1 proteins, which operate as mechanically gated ion channels, allow positively charged ions, including calcium, to flow into the cell (Coste et al. 2010; Wu et al. 2017) are located right at the gastrin release sites.

A very elegant study has recently shown that the EC cells of the small intestine, which release serotonin, express the fast-responding Piezo subtype, namely Piezo2 (Wang et al. 2017). Indeed, Piezo2 is considered as being characteristic for sensory cells, such as Merkel cells in which Piezo2 is the primary mechanosensor (Woo et al. 2014). Apparently, EC cells share several features with Merkel cells, such as a genetic lineage depending on the transcription factor Atoh1 (Yang 2001; Wright et al. 2015) and the secretion of serotonin (Forsberg and Miller 1983; Tachibana et al. 2005). The histochemical figures presented by Wang et al. (2017) make it clearly visible that, in EC cells, the Piezo2 immunoreactivity is also primarily located at the basolateral region of the cells. Furthermore, the functional analyses by Wang et al. (2017) demonstrated that an activation of Piezo2 channels by force is coupled to the secretion of 5-hydroxtryptamine. Based on the similarities, we consider it conceivable that Piezo1 channels have similar implications for the release of gastrin from G cells in response to mechanical stimuli. However, since Piezo channels appear, in addition to mechanosensitivity, to be implicated in a variety of other functions (see Alper 2017), other effects of Piezo1 in G cells cannot be ruled out.

The finding that, in the spindle-like G cells, which extend long processes parallel along the vertical invaginations, Piezo1 is located primarily in the cell bodies but not in the processes is of particular interest, as the hormone gastrin also appears to be concentrated within the cell bodies (Fig.7). Thus, both gastrin and mechanosensitive Piezo1 protein also seem to be segregated in a distinct compartment of this unique cell type. These observations suggest that the longitudinal G cells release the hormone only from the cell soma region. Any kind of functional implications of this segregated distribution of gastrin and Piezo1 is elusive. One speculation is based on the structural similarity with muscle spindles, which are defined as small spindle-shaped cells located in skeletal muscle tissue and that extend parallel to the main muscle fibers. They are stretch receptors that register changes in muscle length and the mechanosensory registration of changes in cell length occurs in the middle part of the cell (by the dendrites of Ia fibers). Whether these structural analogies can be considered as a hint for the possible role of the spindle-like G cells in sensing changes in the length of the invaginations have to be assessed in further studies.

The results of this study suggest that mechanosensitive channels, such as Piezo1, which are located at the exocytic release sites for the hormone, enable G cells to respond directly to antrum distensions with the secretion of gastrin.
